# Precancerous Lesions in Colorectal Cancer

**DOI:** 10.1155/2013/457901

**Published:** 2013-05-14

**Authors:** Fayez Sandouk, Feras Al Jerf, M. H. D. Bassel Al-Halabi

**Affiliations:** ^1^Gastroenterology Specialty Center, Damascus, Syria; ^2^Syrian National Cancer Registry, Office in Charge, Damascus, Syria; ^3^Lab of Human Genetics, Molecular Biology and Biotechnology Department, Atomic Energy Commission of Syria (AECS), Damascus, Syria

## Abstract

Colorectal cancer (CRC) is the third most common cause of cancer death in the world. The incidence rate (ASR) and age distribution of this disease differ between most of African-Middle-Eastern (AMAGE) and North America and Europe for many reasons. However, in all areas, “CRC” is considered as one of the most preventable cancers, because it might develop from variant processes like polyps and IBD in addition to the genetic pathogenesis which became very well known in this disease. We tried in this paper to review all the possible reasons of the differences in incidence and age between the west and AMAGE. Also we reviewed all the mutations that lead to the hereditary and familiar clustering of this disease with the correlations with the surrounding food and environment of different areas. Then, we focused on the precancerous pathology of this disease with special focusing on early detection depending on new endoscopy technology and most important genetic studies. We lastly reviewed the evidence of some of the surveillance and put suggestions about future surveillance programs and how important those programs are on the psychological aspect of the patients and their families.

## 1. Introduction

There are many cases of cancer in gastrointestinal tract which are arisen from a precancerous (premalignant) lesions. This brings a window of opportunity for both scientists to look at the steps of development of cancer and also for patients to be scheduled for surveillance procedures and to get their cancer diagnosed earlier, and subsequently having earlier eradication.

This fact is very clear indeed in colorectal cancer “CRC” which is considered as one of the most preventable cancers, because it might develop from variant processes like polyps, which can be removed before they become cancerous [1], a process that usually takes 10–15 years [2]. In addition to that, most of the mutations that lead to the hereditary and familiar clustering in CRC also became well known leading to easier tracing for the related families [3–6]. Lastly, chronic inflammatory bowel diseases (IBD) especially chronic ulcerative colitis “CUC” have been also documented in “increased risk” category to develop CRC [7].

In this paper we will review all those three facts, focusing on the steps in malignant degeneration, mentioning the difference between the west and the middle east/Africa “AMAGE,” review the evidence of some of the surveillance programs in those two areas of the world, and lastly put suggestions about future surveillance programs. 

## 2. Epidemiology: Incidence and Age (Special Focus on the Young Age in AMAGE)

**Figure 1:**
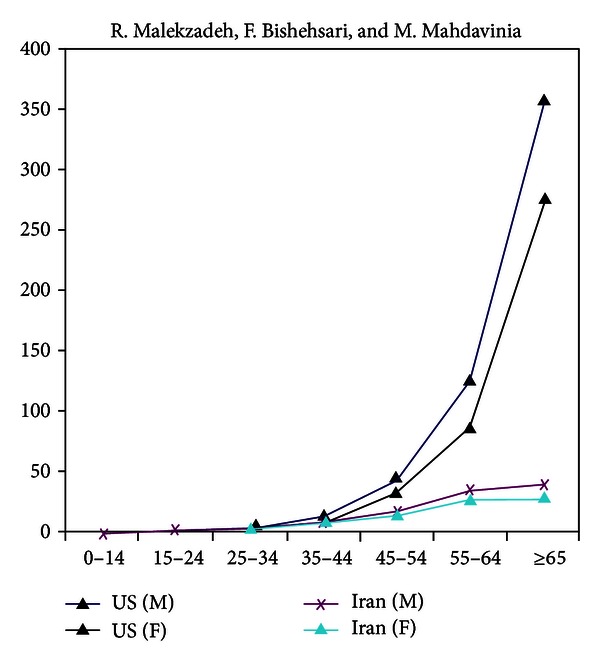
A comparison between the colorectal cancer age-adjusted rates per 100,000 in Iran and the US. The US rates have been calculated from the five-year age-specific SEER data from 1995 to 1999 and age structure of the US during the same years [29].

Colorectal cancer (CRC) is the third most common cause of cancer death in the world [8]. It has 150,000 new cases and 50,000 deaths annually inside the United States [9]. An estimated 148.810 people were diagnosed in 2008 in USA, and about 49,960 people died from the disease. The incidence rate (ASRs) in North America and Europe is approximately 30–50/100.000 [10], this *incidence* is markedly less in most African-Middle-Eastern (AMAGE) countries ranging between 3–11/100.000 [11–17]. This low incidence is may be attributable to certain factors such as the young population of the screening section, high intake of fruits and vegetables, and the life style of the people in those countries [18]. Another difference in the behavior of CRC between the west and AMAGE is *the age* distribution. CRC is thought of as a disease of older people, with more than 90% of patients being diagnosed after the age of 55 years in the West and USA [19], and at the time we find CRC below 40s between 2% and 8% only there [20–26], it is striking that it is a much higher percentage in Syria for example, where it reaches around one-third (23%) [14]! This proportion is close to those seen in many other Middle-Eastern countries like Iraq, Saudi Arabia, Sudan, Morocco, and Iran [12, 13, 27, 28], see Figure 1.

In Egypt, where the proportion of young cases is exceptionally high (36%), a genetic predisposition is more likely to be added, and one should think there of a unique pattern seen at the molecular level [30]. However; one should be careful when talking about this correlation between incidence and age. Iran is considered a country with low-risk of CRC and polyps for older individuals in particular [31, 32]; however, one can appreciate the high proportions of young CRC cases. This is most probably due to the young age structure there. The patients' median age in most surveys in Iran and many neighboring countries is around 52 years, compared to 65 years in the USA for example, and in some areas in AMAGE it might reach only 4% of population whom are more than 65 years [13, 21]! The reason may be because younger patients are more likely to present at tertiary centers than elderly ones who may not present to hospital due to other morbidities or economic considerations and therefore may never receive a cancer diagnosis, despite dying from CRC [33]. A third observation in CRC behavior in the last decades is that its incidence in US and Europe has been *declining* since the mid-1980s, with accelerating drop in recent times, largely due to an increase in screening among individuals aged 50 years and older [21]. In our “AMAGE” regions however the incidence is rising, which may be due the insufficiency in the campaigns and/or due to the “westernization” of the life style during the past three decades with significant lifestyle changes into sedentary shape and the diets became rich in fat and meat, with poor cereals and fibers typical of Western population [34–36] as specifically recognized in Saudi Arabia [30] and Jordan [37]. One last observation in CRC is the variation in *location* between the West and AMAGE countries as we see carcinomas and polyps more frequently in the left colon in the West meanwhile, we notice considerable right colon carcinomas in Iran especially in young and HNPCC (Lynch) patients [29, 38].

## 3. Pathogenesis: Molecular Genetics, Diet Relationship, Syndromes, Prognosis, the Role of Genetic Testing in CRC, Races and Habits Variations, and IBD

**Figure 2:**
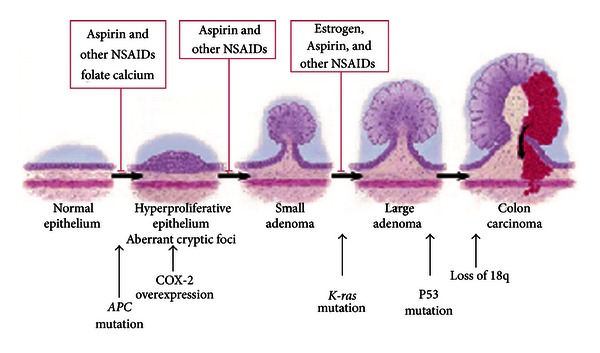
Explaining CRC development.

Generally speaking, 50%–90% of all CRCs are sporadic and 10%–50% is hereditary or familial clustering in etiology. In Sporadic CRC, somatic mutations in *APC*, *K*-*ras, *and p53 were proposed to act sequentially in progression from normal mucosa to carcinoma in CRC [39] by causing errors in the way of “grow and repair mechanism” of the cells lining the colon (Figure 2). It starts by hyperproliferation in the normal epithelium and aberrant cryptic foci forming small adenoma, after that the K-ras mutation will increase the size into large polyp, and lastly with the combination of p53 mutation and the loss of 18q, malignant cells will appear forming the colon carcinoma.

This process can be blocked by NSAIDs, Aspirin, Calcium, Folate and Estrogen. A recent study showed that a total of 189 “candidate” cancer genes can alter in sporadic CRC [40]. These new findings indicate that CRC, in addition to the environmental background, is a more complex genetic disease than what was anticipated in the past, and thus studying and comparing CRCs from populations with different epidemic features of the disease can help us better understand the underlying mechanism of CRC carcinogenesis [41]. 

### 3.1. Diet Relationship

The source of most of the molecular data on the molecular alterations in CRC has come mostly from studies on Western populations, and little information is available on molecular features of tumors from the AMAGE countries. A study from Italy showed that there was a marked mutation spectra, namely, the G to A transition in the second base of codon 13 of K-ras in patients with CRC [42]. This has been previously shown to be correlated with high consumption of refined grain36 which is a dietary pattern directly associated with the CRC risk [43]. This mutation showed only minimal changes in another study from Iran where they use wheat-based breads with a variety of unrefined, unleavened whole-wheat breads [44]. Another study in Iran showed a positive association between high serum folate/vitamin B12 levels with promotor methylation of tumor-related genes and risk of CRC association with particular polymorphisms called “the methylenetetrahydro folate reductase gene” (*MTHFR *C677T) and not all the genotypes [45]. The authors concluded that caution should be practiced in the mandatory fortification of cereals with folic acid, and vitamin supplementation should be considered to decrease the risk of sporadic CRC. 

### 3.2. Syndromes

CRCs which have genetic predisposition like HNPCC or MSI were confirmed to be associated with a specific type of mutation in *K-ras* both in West and AMAGE [46]. However, some difference was noted in the mutations of the DNA mismatch repair (MMR) genes of HNPCC (Lynch) as it was 4.7% of probands in Iran, which is relatively high in comparison with most Western populations, reporting a frequency of 1%–3%. The result of this study would give us an insight of the prevalence of MMR gene mutations in Iranian HNPCC which could be used for implementing preventive strategies in familial CRCs [29, 47]. Another important hereditary condition is the juvenile polyposis syndrome (JPS) which is inherited as an autosomal dominant due to specific mutation in the *BMPR1A *gene or the *SMAD4 *gene. In this syndrome, more than 3–5 juvenile polyps are seen in the colorectum in addition to the stomach of the small intestine too [48–50], and there is an obvious increase for developing CRC in 9% to 50%, and also of gastric, small intestine and possibly pancreatic cancers although Brosens et al. reported no upper gastrointestinal malignancies in their cohort [49–51]. It is wise here to remind that this syndrome differs from the benign single juvenile polyp that is usually seen in the rectum of children and does not need any follow up [52, 53].

### 3.3. Prognosis

Finally, molecular markers in CRC such as mutations, polymorphisms, chromosomal alterations, protein expressions, and growth factors have been shown to significantly influence the disease progression and/or response to therapy. A study from Iran showed that locally advanced rectal cancer treated with radiotherapy has shown that epidermal growth factor receptor (EGFR) expression may serve as a predictor of pathologic response to treatment and may benefit from more therapeutic modalities [54]. Another two large retrospective reports on CRCs tried to identify the prognostic factors focusing on clinical characteristics of the tumors [55, 56]. Although it was shown p53 mutation has significant role in CRC progression independent of the population, and a specific type of it like CpG n.213 is very distinct for the proximal CRC [57]. There are other studies from Egypt, Iran, and Syria which showed that familial clustering of CRC is more frequent in younger probands and right-sided tumors, and others showed that the location was of no real significance in the disease prognosis [14, 55, 56]. 

### 3.4. The Role of Genetic Testing in CRC

As mentioned before; CRC results from interactions between genes and environment, and sporadic CRC accounts for about 80% of all CRC, while the hereditary accounts for the rest. The most known causative genes for genetic CRC that have been cloned and characterized within the past decade are those with polyps like familial adenomatous polyposis FAP, AFAP, juvenile polyposis, Peutz-Jeghers syndrome, Cowden syndrome and with no polyps (hereditary Nonpolyposis CRC, HNPCC). Individuals have an increased risk of CRC when they have one additional first- or two additional second-degree family members with CRC, regardless of age at diagnosis or when we have early ages at onset were patients in whom cancer was diagnosed under the age of 45 years [58, 59].

Genetic testing is being adapted increasingly to identify individuals with germline mutations that predispose to hereditary colorectal syndromes. Deciding who to test and for which syndrome is of concern to members of the GI oncology community, molecular geneticists, and genetic counselors. 

### 3.5. Genetic Testing and Mutations Analysis

Before getting into the genes, DNA should be extracted and then amplified by the polymerase chain reaction PCR into many separate segments, after that, the mutations are identified.

#### 3.5.1. Methods of DNA Extraction

Genomic DNA was prepared from anticoagulated (EDTA) venous blood samples, and tumors tissue from unrelated Syrian patients using standard techniques, employing Qiagene Kit (Qiagen). The method of the DNA amplifying is achieved by the PCR into 32 separate segments using primers for the entire coding region of the APC gene and exons 7 and 13 of MUTYH (which harbor the two common MUTYH mutations G382D and Y165C) [60, 61]. 

### 3.6. Molecular Analysis of Small Mutations

More techniques were used to analyze different kinds of mutations in the APC gene and MUTYH gene and other genes (Table 1) [62–64]. 

### 3.7. Denaturing High-Performance Liquid Chromatography (DHPLC)

DHPLC is a sensitive technique with high throughout-put to detect small mutations (small deletions or insertions leading to frame shifts, splice site alterations, and nonsense and missense mutations) in DNA fragments with length of 100–500 bp. The exons 1 to 14 of APC gene, exons 7 and 13 of MYH, each of them in the range of 100–400 bp, exon 15 APC gene, and exon 7–13 MUTYH were analyzed by DHPLC (WAVE system, transgenomic) [65]. 

### 3.8. Sequencing of Genomic DNA

APC is a tumor suppressor gene, and inactivation of it leads to neoplastic tumor growth [66]. The most commonly used molecular test is the protein truncated test (PTT) which detects up to 80% of the APC mutations [66]. Other tests such as the in vivo yeast fusion protein assay may be also used as an alternative test to PTT [66]. DNA sequencing is then performed to characterize the germline mutation. This APC mutation gene test is 100% accurate in the affected first-degree relatives.

Automated sequencing of the entire coding region of APC and analysis of the 2 most prevalent MUTYH gene mutations (G382D and Y165C) revealed pathogenic mutations in the APC or MUTYH genes in CRC families (FAP-AFAP) [2]. This APC gene testing is cost effective when compared to flexible sigmoidoscopy until age 60 [67]. If the first-degree relative does not carry the APC gene, then the patient himself is not a high risk for FAP and no need for surveillance sigmoidoscopy. However there is 26% of those APC gene-negative carriers still doing the sigmoidoscopy as they do not trust this molecular technology in the clinical practice [67]. Moreover; molecular gene test can be used as a guide to *surgical* management of FAP patients [68] as prophylactic colectomy is considered as a part of the surgical management because of the known adenoma-carcinoma sequence [68]. APC mutations in the APC gene 3* of codon 1250 are associated with an increased risk for rectal cancer, and subsequently the surgical preferable choice for patients with such mutations is the restorative proctocolectomy with ileal pouch-anal anastomosis [68].

### 3.9. Biological Analyses

Sequence analyses were performed by BioEdit software. For the changed base sequences, the mutational site and type were determined in NCBI, and then the mutations were identified as new by referring to the mutations reported in the human gene mutation database (http://www.hgmd.cf.ac.uk/ac/gene.php?gene=APC) and the UMD-APC mutations database (http://www.umd.be/APC/).

### 3.10. Molecular Analysis of Large Rearrangements Detection

*Multiplex ligation-dependent probe amplification (MLPA)* was used to screen DNA samples from CRC patients, for large genomic rearrangements of APC (large fragment deletions) without micromutations according to the manufacturer's instructions (SALSA MLPA KIT P043 APC kit, P003-MLH1/MSH2 Kit, P008-A1 MSH6/PMS2 Kit, MRC-Holland, Amsterdam, the Netherlands) [69–72]. The MLPA method is based on sequence-specific probe hybridization to genomic DNA, followed by polymerase chain reaction (PCR) amplification of the hybridized probe (with one amplification primer fluorescently labeled), and semiquantitative analysis of the PCR products. Target-specific products are identified according to their differential length. MLPA reactions were analyzed on an ABI 310 sequencer using Genotyper software, version 3.0 (Applied Biosystems, Foster City, CA, USA). And Coffalyser program for normalization and relative probe signals were calculated by dividing each measured peak area by the sum of all peak areas of the sample. The ratio of each individual relative probe area was then normalized to the mean obtained with three control samples.

### 3.11. Molecular Analysis of Microsatellite Instability in Colorectal Cancer

Factors such as gene understanding, cumulative absolute risk for CRC, clinical criteria for hereditary cancer, and specific genotype-phynotype correlations need to be evaluated when molecular genetic testing is to be used for preventive or therapeutic management of those affected individuals and their high risk relatives. 

*Microsatellite instability (MSI) is the molecular fingerprint of a deficient mismatch repair system.* Approximately 15% of colorectal cancers (CRCs) display MSI owing either to epigenetic silencing of MLH1 or approximately 2%-3% of all CRCs which are caused by germline mutations in one of the mismatch repair genes (MLH1, MSH2, MSH6, and PMS2). Methods to detect MSI are well established and routinely incorporated into clinical practice. A clinical and molecular profile of MSI tumors has been described, leading to the concept of an MSI phenotype in CRC [73, 74]. CRC is the result of accumulation of multiple genetic mutations, occurring over a period of 10–15 years. Microsatellite instability associated to DNA mismatch repair genes (MMR) mutations account for 20% of all CRC cases and 85% of hereditary CRC [75]. The role of MSI as a genetic marker of Lynch syndrome is well established in the clinic. Both MSI detection and immunohistochemistry are highly sensitive methods for the identification of individuals with defective MMR molecular techniques developed during the past two decades allowing reliable detection of MSI.

At last, it is nice to mention that the current findings suggest that hopefulness may predict resilience after HCRC genetic testing in Hong Kong, China. Interventions to increase the level of hope may be beneficial to the psychological adjustment of CRC genetic testing recipients [76]. 

In summary, the genetic consideration for a family with features of the Lynch syndrome are microsatellite instability testing, consideration for immunohistochemistry evaluation, and the MMR mismatch repair gene testing, while, in contrast, a patient with FAP will require *APC* testing, and at last other germline mutations yet to be also identified when proved. 

In the pathogenesis issue of CRC, one should also pay attention to the *races and habits variations.* Differences in dietary and environmental exposures must be considered upon a background of genetically determined susceptibility. This might explain the higher incidence of CRC in younger CRC incidence rates in shared environmental exposures in economically transitioning countries [77] & countries with limited resources including Mexico and Brazil in South America and Romania and East Europe [78]. The substantial decrease in physical activity, excess energy intake, and change in dietary habit might now be reflected in younger population who shared these exposures during childhood and younger adulthood which suggest a genetic predisposition [29]. An unfavorable diet such as consumption of red meat and processed meat in children and young adults over the past three decades may have contributed to this [79]. Special care must also be considered in specific areas with special environmental events like the blame of exposure to chemical and radioactive carcinogens of wars in the recent past in the Kurdish region of Iraq [27]. In Syria, we noticed that the highest rates of cancer and CRC in specific were in Tartous, the second coast city, where there are a lot of plastic greenhouses wondering if there is any relationship [14]. Lastly, few articles from Japan reported cases of carcinoma of the colon as a complication of long standing colonic Schistosomajaponicum infection; however, development of colonic malignancy with Schistomamansoni has not been established [80–83].

The last important subject in the precancerous lesions for CRC is the chronic *inflammatory bowel disease* which is considered as an Increased risk category disease for the development of colorectal cancer in approximately 7% to 10% at 20 years of disease and as high as 30% after 35 years of disease [6]. This risk depends on several factors, the most important is the duration, as we mentioned, and also the extent of the disease. Other risk factors include PSC, family history of colon cancer, age at diagnosis of disease, severity of inflammation, and possibly backwash ileitis [84–89]. The IBD-associated pseudopolyps by themselves are not dysplastic and are not at a risk factor for colon cancer. However, their presence can complicate the recognition of true adenomas and dysplastic associated lesion mass (DALM). Resection of these polyps is not necessary and biopsies should be taken from the base and the inflamed mucosa in between. Once IBD-associated CRC becomes symptomatic, the malignancy is usually advanced, and the prognosis is correspondingly poor [90]. 

Indeed, the complexity of the carcinogenesis makes it difficult to depend only on the clinical characteristics in the prognosis. Therefore, prospective studies along with studies of molecular markers in CRC are necessary for predicting the course of the disease and choosing the appropriate treatment.

## 4. Precancerous Pathology in CRC, Recent Endoscopic Technology, and Early Resection

**Figure 3:**
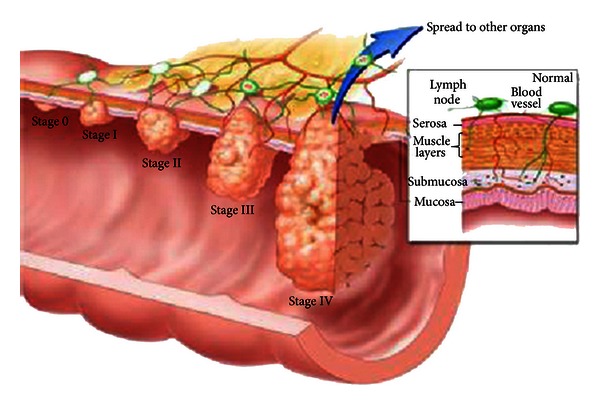
Stages of CRC.

After forming carcinoma, there are five stages for the disease (Figure 3), stage 0 where the tumor locates in the mucosal layer of colon, stage I when it reaches the muscularis layer, stage II when it just perforates the serosa, stage III when the surrounding lymph nodes are involved, and lastly stage IV with distal metastasis. The ideal step in CRC screening is to discover it in the precancerous lesions and also up to stage I when we can resect it endoscopically, and in order to achieve this, we should seek better technology to discover earlier lesions.

Three percent to six percent of all colorectal cancers have been overlooked in conventional videoscope in retrospective studies [91] usually due to incomplete examination, insufficient bowel preparation, incompetence of endoscopists, incomplete polypectomy, and lastly mucosal flat lesions especially in right colons. As we can improve the yield of the first four reasons by endoscopy training programs, it is not the same in the last one. We need to use extra modalities like chromoendocopy to appreciate the small flat lesions, and more recently we can use the high definition (HD) digital chromoendoscopy systems (Pentax i-Scan, Olympus NBI and Fujinon Fice). These technology accentuate suspicious mucosal structures are giving us better delineation of borders and better vasculature pattern by the enhancement of minute mucosal and vessel structures, and sometimes with the superficial enhancement technology it can give us ideas about the cytological pathology specially when we add magnification property. Heibig et al. showed that by using the high definition (HD) digital chromoendoscopy technology we can improve the prevalence of missed adenomas from 58% in standard video scopes to 15% only in HD systems [92] although Professor Shim from Korea had recently reported negative results in this concern [93]. Anyhow, every one agrees that these new technologies became very helpful giving a better idea about the characteristics of mucosal lesions from the benign or malignancy point of view. 

Morphological classification into *Polypoid, Flat and depressed* types is now rather sensitive indication for malignancy. In two excellent studies showed that the Prevalence of CRC in depressed, nonpolypoid lesions were between 23.6–35.9% meanwhile it was around 1.4–1.7% only in the polypoid and flat lesions [94]. 

Another good classification depends on the *Glandular type* of the lesions (Modified Kudo's “pit” pattern Classification) [95] where adenomas were divided into five types graded from I to IV. In one good study, there were only two malignant lesions out of 65 of type I & II, meanwhile all the 80 lesions of type III through type V were neoplastic reflecting 97% sensitivity and 100% specificity [95]. Special training centers are available now in AMAGE area for young doctors and addresses for which are available with the authors for the interested people. 

## 5. Surveillance Campaigns and Future

The extreme goals of cancer surveillance and prevention programs are to identify those patients at high risk of developing a certain cancer, in order to treat the precursor condition, discover the precancerous lesions at a very early stage, discover new findings in tumor's markers studies, and then widespread the available therapeutic options for prevention at the genetic, pathologic, and endoscopic levels. 

*“A penny of prophylaxis is far much better than bunch of treatments”* is a golden principle that can be a title for all campaigns for any danger including CRC. In 2009, we did a rapid website search which surprisingly showed 246,000 sites under the title “Colorectal Cancer Campaign” and 158,000 sites under the title “Prophylaxis of Colorectal Cancer” including one of the home pages of the BBC News at that year! In West, there are lot of campaigns programs that proved their prophylactic efficiency and efficacy. A book with a title “Are Public Awareness Campaigns Effective?” by Lacey Meyer showed that such campaigns can reduce 70%–90% of the CRC incidence [96]. On the other hand, in AMAGE region the rate of screening is very low globally and may be negligible which is usually due to cost and resistance by physicians, patients, and the healthcare system [29]. 

As we mentioned before, the most important step in such programs is defining the high risk group of people for CRC. The National Comprehensive Cancer Network (NCCN) [47] had nicely classified those in details into three groups. *The average risk group* which includes age above 50, with no personal or family past history of colonic polyps, CRC, or IBD. In this category of people, a surveillance colonoscopy after 50 years of age is to be repeated in 5 years, and then after 10 years. *The increased risk group* which includes positive personal or family history for adenomatous sessile/serrated polyp, CRC, or IBD for over 10 years. Here, they divide the cases into low risk patients when we have 1-2 polyps history, less than 1 cm in size and tubular type of polyps, where a surveillance colonoscopy is to be done after 5 years. A moderate risk patients are when there are 3–10 polyps, villous type with moderate or high grade dysplasia, where the surveillance colonoscopy is to be done after 3 years and when there are more than 10 polyps where polyposis syndromes should be considered, and to be screened accordingly. When we have a suspicion of incomplete resection of big polyps, repeated colonoscopy is to be done in 3–6 months; however, when we diagnose tumor in situ, a surveillance colonoscopy is recommended after 3–6 months of resection then every 1 year for 5 years to be repeated every 3–5 years whether polyps were found or not. As for the relatives, a surveillance colonoscopy is to be done at the age of 10 years less than the CRC onset in the patient. *Lastly,* we have* the high risk group* which includes the variant types of syndromes. The first is the nonpolyposis (hereditary nonpolypoid colon cancer (HNPCC) with its two types: Lynch I & II), which is an autosomal dominant disease that occurs due to mutations in the DNA mismatch repair (MMR) genes MLH1, MSH2, MSH6 and PMS2 which is known to be positive in around 1%–3% in the West, meanwhile it is 4.7% in Iran for example! Therefore, studying the microsatellite instability (MSI) in the family members of CRC might be considered in the CRC campaigns in AMAGE area [3, 4, 47, 97, 98]. familial adenomatous polyposis (FAP) is the most common and it is an as autosomal dominant transmission due to mutation in the APC genes, and the endoscopy picture is so typical of hundreds of polyposis. We do the APC gene, and when it is positive a very close surveillance colonoscopy is recommended every year till 24 of age, then every 2 years till 34 of age, every 3 years till the age of 44, and lastly every 3 years afterwards. The other example is MYH-associated polyposis syndrome where mutations are in the base-excision repair (MYH) genes that transmitted in an autosomal recessive inheritance and appear in older age than FAP and associated with duodenal polyps too. Here, we do the MYH mutation and if it is positive, then we do surveillance colonoscopy and duodenoscopy every 1-2 years meanwhile it is done every 2-3 years in the negative MYH mutation. All other polyposis syndromes can be easily traced in this article [47]. At last and over the past three decades, various colonoscopic surveillance programs in ulcerative colitis (UC) have been reported and the details of these programs have differed according to patient selection, endoscopic protocol, management strategies, and the experience of the centers. The results of most of those programs have shown that CRC which is diagnosed at an early stage has rather good prognosis, and the majority were able to safely retain their colons. On the other hand, the development of advanced carcinoma was not always prevented, and in some series, the yield of neoplasia was so low that the value of the program was questioned. Prophylactic proctocolectomy avoids CRC, but is not justified in patients whose health is otherwise good due to significant postoperative morbidity [99]. Colonoscopic surveillance has been advocated as an alternative means to reduce cancer-related mortality among individuals who wish to retain their colon. The standard protocol for this kind of surveillance is to do colonoscopy with 4 biopsies each 10 cm while withdrawing the scope starting from the caecum searching for dysplasia lesions [100]. No randomized study has ever been conducted to substantiate the efficacy or cost effectiveness of colonoscopic surveillance in patients with UC or Crohn's disease (CD); however, various case-control studies provide indirect evidence supporting its efficacy in UC on the basis of improved cancer-related outcome. Specifically, these studies have shown surveillance in UC which is associated with an earlier stage of cancer diagnosis [75, 87–89], improved cancer-related survival [101–104], and reduced overall death rates [90]. It was also found that surveillance colonoscopy helps especially when the initial biopsies were negative for dysplasia; on the contrary when the initial biopsies were positive for dysplasia where usually presented later on with advanced picture of CRC where total colectomy is may be favorable [100]. The other case when total colectomy is recommended in CUC is with patients with extensive, left-sided disease which share the same risk of the development of dysplasia [100]. In addition, theoretical modeling analysis using a computer cohort simulation evaluating 17 different surveillance strategies predicted an improved life expectancy in patients undergoing surveillance. These combined data have resulted in qualified support for surveillance in UC by a Cochrane analysis [105]. Data concerning the value of colonoscopic surveillance in *Crohn's* disease CD are extremely limited. One series from a private practice in New York studied patients with extensive Crohn's colitis (affecting more than 30% of the large bowel) for more than eight years duration. Sixteen percent were diagnosed with neoplasia, half of which were detected at the time of initial screening investigation [106]. Another small case control study found a protective effect on CRC risk if a colonoscopic examination was conducted for cancer screening or surveillance purposes in Crohn's colitis (odds ratio of 0.21, 95% C.I. 0.04–0.77; *P* = 0.02) [107].

There is still a rather difficult question to answer in AMAGE area concerning the surveillance colonoscopy, how can we convince female clients with screening colonoscopy and how can we overcome restrictions? Here was the role of the public awareness (PA) campaigns. The other way of surveillance screening is doing the stool occult blood which has rather less sensitivity than colonoscopy and there is no need for it if colonoscopy is to be done; however, this test might be useful in the high risk patients in countries with short resources. 

In fact, such campaigns are not only colonoscopy or OB screening, but they are global PA projects that are supposed to involve a wide range of aspects including the patients, families, healthcare workers and providers, family doctors, endoscopists, resources, and media and also should take in consideration the special surrounding environmental events like pollutants, depleted uranium, chemicals, and so forth. 

The British society guidelines favor segmoidoscopy with OB testing and for the positive tests they go for colonoscopy [69, 70], and the American cancer society 2011 starts the surveillance colonoscopy at 5 years or at the age from 10 years below the CRC event in the family [71, 72]. 

Because there is high CRC incidence in young below 40 years, the Korean screening program starts at age of 40 [108], the same like Syria where we do surveillance colonoscopy on the related family starting from 40 years or when we have a history of obviously seen blood in the stool starting at 5 years of age; however, if there was no history of seen blood in stool we do the OB every year starting at age 5 and refer the patient for colonoscopy when OB became positive [14]. This age of 40 has already been reported as the standard time to start the surveillance in most of the countries in AMAGE like Iraq, Saudi Arabia, Sudan, Iran, and Egypt [12, 13, 27, 28, 30]. 

Of course these guidelines are changeable and differ according to geographic regions. The American Cancer Society recommends exercising five or more days a week for at least 30 minutes a day and they encourage eating a nutritious diet [109]. A study from Abu Dhabi showed that getting enough exercise and controlling body fat could prevent 45% of colorectal cancers and they recommend a low-fat diet that includes plenty of fibers and at least five servings of fruits and vegetables per day, discouraging a diet high in red, processed, or heavily cooked meat and they recommend to cut down or stop smoking and alcohol [109]. 

At last, as we in AMAGE have almost similar environment and life habits, we should try for future prospects to focus in our surveillance guidelines on the following.

1. Focus on the age-specific incidence rates and study the mortality and survival rates and decide the composite stage of the disease at the time of initial diagnosis to understand the details of the behavior of CRC in our area.
2. We need international collaboration & extensive studies at clinical and molecular levels to discover the CRC carcinogenesis mechanisms and find sensitive markers for early diagnosis and subsequently more effective therapy
3. Promote public awareness and screening strategies in those families with a member affected by CRC especially at younger age or with proximal tumors.
4. Educate general practice and family physicians about the available prevention options like using Aspirin and other NSAIDs, Folate Calcium, and Estrogen to cut the pathology pathway from adenoma toward cancer (Figure 2).
5. Encourage healthcare providers for early awareness.
6. Support whole-grain foods diet with Unrefined grain which has been shown to reduce the risk of several types of neoplasms, particularly of the digestive tract including CRC [110], to minimize the chance of carcinogenic K-ras mutation which happens from the refine grain such as white bread, rice, and pasta [46] which should be prevented by educating the public about their hazards.

## 6. Psychological Aspect

One last subject that we think helps in supporting the preventing CRC campaigns is dealing with the psychological aspect. Although genetic counseling was not found helpful in altering the level of perceived risk and anxiety, but also it definitely improve knowledge of cancer genetics and this might make pooled short-term difference [111]. Also, it was found that surveillance colonoscopy following attendance at colorectal screening using flexible sigmoidoscopy had great reductions in anxiety, and the overall surveillance itself appeared to be associated with better psychological wellbeing that comes from getting rid of cancer worry rather than being assigned to colonoscopic surveillance per se [112]. Another study from Sweden however showed that most of the patients did not demonstrate increased levels of anxiety or depression towards the surveillance colonoscopy program [113]. One excellent book by Susan L. Gearhart from John Hopkins (Early diagnosis and treatment of cancer) collected many real patients who had made themselves determined to recover and felt that having a positive attitude had been helpful especially after talking openly about their disease and the treatment they had undergone. Participants also found learning to live with cancer seems to involve both accepting the situation and finding the inner resources to cope [114]. 

## 7. Conclusion

CRC is one of the most preventable cancers in the world because we have already known lots of knowledge on the genetic pathogenesis of this disease and correlations with the surrounding food and environment to the degree that we can interfere with many useful precaution plans. The established precancerous lesions of this disease like polyps, dysplasia, and IBD also give us better opportunity for earlier discovery with subsequent resection. International and regional collaboration is highly recommended to reach the ideal public awareness campaign to protect us from this disease.

## Figures and Tables

**Table 1:** Proposed categories of hereditary colorectal cancer syndromes for which genetic testing is possible.

Categories of hereditary colorectal cancer						
	FAP	AFAP	HNPCC/Lynch	Juvenile polyposis	Peutz-Jeghers	Cowden syndrome
Mean age at diagnosis of CRC	32–39	45–55	42–49	34	64	20
No of polyps	>100	10–100	1 (ie tumors)	5–10 or more jp	Two or more PJ polyps	
Gene	*APC **MYH**	**APC** **MYH **	**MHS2**, **MLH1**, **PMS1**, **PMS2 **	**SMAD4** **BMPR1A**	**STK11/LKB**	**PTEN **
Mode of inheritance	AD	**AD ***AR		AD	AD	AD

*Adenomatous polyposis Coli.

**Autosomal dominant.

***Autosomal recessive.

## References

[2] Vogelstein B, Fearon ER, Hamilton SR (1988). Genetic alterations during colorectal-tumor development. *The New England Journal of Medicine*.

[3] Kinzler KW, Vogelstein B (1996). Lessons from hereditary colorectal cancer. *Cell*.

[4] Lynch HT, de la Chapelle A (2003). Hereditary colorectal cancer. *The New England Journal of Medicine*.

[5] Vasen HFA (2007). The Lynch syndrome (hereditary nonpolyposis colorectal cancer). *Alimentary Pharmacology and Therapeutics*.

[6] Grady WM (2005). Molecular basis for subdividing hereditary colon cancer?. *Gut*.

[7] Lewis JD, Deren JJ, Lichtenstein GR (1999). Cancer risk in patients with inflammatory bowel disease. *Gastroenterology Clinics of North America*.

[8] Parkin DM (2001). Global cancer statistics in the year 2000. *The Lancet Oncology*.

[9] Hutfless S, Kalloo AN (2013). Screening colonoscopy: a new frontier for nurse practitioners. *Clinical Gatroenterology and Hepatology*.

[10] Schottenfeld D, Winawer SJ, Schottenfeld D, Fraumeni JF (1996). Cancers of the large intestine. *Cancer Epidemiology and Prevention*.

[11] Parkin DM, Whelan S, Ferlay J, Teppo L, Thomas DB (2002). *Cancer Incidence in Five Continents*.

[12] Stewart BW, Kleihues P (2003). *World Cancer Report*.

[13] Incidence des cancers a Rabat 2006–2008.

[14] Ansari R, Mahdavinia M, Sajadi AR (2005). Incidence and age distribution of colorectal cancer in Iran: results of a population-based cancer registry. *Cancer Letters*.

[15] Al Jerf F, Syrian National Cancer Registry The problem in ministry of health in Syria is that they have not put those data in the websites until now.

[16] Ministry of health: results of Iraqi cancer registry.

[17] Ayyub MI, Al-Radi AO, Khazeindar AM, Nagi AH, Maniyar IA (2002). Clinicopathological trends in colorectal cancer in a tertiary care hospital. *Saudi Medical Journal*.

[18] Rasul KI, Awidi AS, Mubarak AA, Al-Homsi UM (2001). Study of colorectal cancer in Qatar. *Saudi Medical Journal*.

[19] Jullumstrø E, Wibe A, Lydersen S, Edna TH (2011). Colon cancer incidence, presentation, treatment and outcomes over 25 years. *Colorectal Disease*.

[20] Heys SD, O’Hanrahan TJ, Brittenden J, Eremin O (1994). Colorectal cancer in young patients: a review of the literature. *European Journal of Surgical Oncology*.

[21] Ries LAG, Melbert D, Krapcho M (2008). *Surveillance Epidemiology and End Results (SEER) Cancer Statistics Review 1975–2005*.

[22] Bulow S (1980). Colorectal cancer in patients less than 40 years of age in Denmark, 1943–1967. *Diseases of the Colon and Rectum*.

[23] Griffin PM, Liff JM, Greenberg RS, Clark WS (1991). Adenocarcinomas of the colon and rectum in persons under 40 years old: a population-based study. *Gastroenterology*.

[24] MacGillivray DC, Swartz SE, Robinson AM, Cruess DF, Smith LE (1991). Adenocarcinoma of the colon and rectum in patients less than 40 years of age. *Surgery Gynecology and Obstetrics*.

[25] Guillem JG, Calle JPL, Cellini C (1999). Varying features of early age-of-onset “sporadic” and hereditary nonpolyposis colorectal cancer patients. *Diseases of the Colon and Rectum*.

[26] Mitry E, Benhamiche AM, Jouve JL, Clinard F, Finn-Faivre C, Faivre J (2001). Colorectal adenocarcinoma in patients under 45 years of age: comparison with older patients in a well-defined French population. *Diseases of the Colon and Rectum*.

[27] Sheet SY, Sheikha AK, Saeed AM, Ameen HA, Mohammed SS, Khasraw M (2012). Colorectal cancer: is the incidence rising in young Iraqi patients?. *Asia-Pacific Journal of Clinical Oncology*.

[28] Isbister WH (1992). Colorectal cancer below age 40 in the Kingdom of Saudi Arabia. *Australian and New Zealand Journal of Surgery*.

[29] Malekzadeh R, Bishehsari F, Mahdavinia M, Ansari R (2009). Epidemiology and molecular genetics of colorectal cancer in iran: a review. *Archives of Iranian Medicine*.

[30] Soliman AS, Bondy ML, Levin B (1997). Colorectal cancer in Egyptian patients under 40 years of age. *International Journal of Cancer*.

[31] Haghighi P, Nasr K, Mohallatee EA (1977). Colorectal polyps and carcinoma in Southern Iran. *Cancer*.

[32] Hosseini SV, Izadpanah A, Yarmohammadi H (2004). Epidemiological changes in colorectal cancer in Shiraz, Iran: 1980–2000. *Australian and New Zealand Journal of Surgery*.

[33] Vakili C, Fatourechi V (1976). Age distribution of patients with carcinoma of the colon in a general hospital in Iran. *Surgery*.

[34] Ferlay J, Bray F, Pisani P, Parkin DM (2004). *GLOBOCAN, 2002: Cancer Incidence, Mortality, and Prevalence Worldwide, Version 2. 0*.

[35] Semnani S, Sadjadi A, Fahimi S (2006). Declining incidence of esophageal cancer in the Turkmen Plain, eastern part of the Caspian Littoral of Iran: a retrospective cancer surveillance. *Cancer Detection and Prevention*.

[36] Parkin DM, Whelan S, Ferlay J, Teppo L, Thomas DB (2005). *Cancer Incidence in Five Continents*.

[37] Al-Jaberi TM, Ammari F, Gharieybeh K (1997). Colorectal adenocarcinoma in a defined Jordanian population from 1990 to 1995. *Diseases of the Colon and Rectum*.

[38] Mahdavinia M, Bishehsari F, Ansari R (2005). Family history of colorectal cancer in Iran. *BMC Cancer*.

[39] Fearon ER, Vogelstein B (1990). A genetic model for colorectal tumorigenesis. *Cell*.

[40] Wood LD, Parsons DW, Jones S (2007). The genomic landscapes of human breast and colorectal cancers. *Science*.

[41] Chan AO, Soliman AS, Zhang Q (2005). Differing DNA methylation patterns and gene mutation frequencies in colorectal carcinomas from Middle Eastern countries. *Clinical Cancer Research*.

[42] Chatenoud L, La Vecchia C, Franceschi S (1999). Refined-cereal intake and risk of selected cancers in Italy. *The American Journal of Clinical Nutrition*.

[43] World Cancer Research Fund/American Institute for Cancer Research (2007). *Food, Nutrition, Physical Activity, and the Prevention of Cancer: A Global Perspective*.

[44] Djazayery A, Pajooyan J (2000). Food consumption patterns and nutritional problems in the Islamic Republic of Iran. *Nutrition and Health*.

[45] Mokarram P, Naghibalhossaini F, Saberi Firoozi M (2008). Methylenetetrahydrofolate reductase C677T genotype affects promoter methylation of tumor-specific genes in sporadic colorectal cancer through an interaction with folate/vitamin B12 status. *World Journal of Gastroenterology*.

[46] Bishehsari F, Mahdavinia M, Malekzadeh R (2006). Patterns of K-rasmutation in colorectal carcinomas from Iran and Italy (a Gruppo Oncologico dell'Italia Meridionale study): influence of microsatellite instability status andcountry of origin. *Annals of Oncology*.

[47] Practice Guidelines in oncology, Version 1. http://www.nccn.org/.

[48] Ley RE, Turnbaugh PJ, Klein S, Gordon JI (2006). Microbial ecology: human gut microbes associated with obesity. *Nature*.

[49] Brosens LAA, van Hattem A, Hylind LM (2007). Risk of colorectal cancer in juvenile polyposis. *Gut*.

[50] van Hattem WA, Brosens LAA, de Leng WWJ (2008). Large genomic deletions of SMAD4, BMPR1A and PTEN in juvenile polyposis. *Gut*.

[51] Brosens LA, van Hattem WA, Kools MC (2009). No TGFBRII germline mutations in juvenile polyposis patients without SMAD4 or BMPR1A mutation. *Gut*.

[52] Sartor RB (2004). Therapeutic manipulation of the enteric microflora in inflammatory bowel diseases: antibiotics, probiotics, and prebiotics. *Gastroenterology*.

[53] Sheil B, Shanahan F, O’Mahony L (2007). Probiotic effects on inflammatory bowel disease. *Journal of Nutrition*.

[54] Motlagh A, Azadeh P, Fazlalizadeh A (2007). Expression of epirdermal growth factor receptor as a predictive factor for rectal cancer. *Archives of Iranian Medicine*.

[55] Mehrkhani F, Nasiri S, Donboli K, Meysamie A, Hedayat A (2009). Prognostic factors in survival of colorectal cancer patients after surgery. *Colorectal Disease*.

[56] Moghimi-Dehkordi B, Safaee A, Zali MR (2008). Prognostic factors in 1,138 Iranian colorectal cancer patients. *International Journal of Colorectal Disease*.

[57] Frigola J, Solé X, Paz MF (2005). Differential DNA hypermethylation and hypomethylation signatures in colorectal cancer. *Human Molecular Genetics*.

[58] Axell L, Ahnen D, Markey K (2005). Basic concepts for genetic testing in common hereditary colorectal cancer syndromes. *Current Colorectal Cancer Reports*.

[59] Terdiman JP, Conrad PG, Sleisenger MH (1999). Genetic testing in hereditary colorectal cancer: Indications and procedures. *The American Journal of Gastroenterology*.

[60] Bougatef K, Marrakchi R, Moussa A (2008). First genetic analysis in Tunisian familial adenomatous polyposis probands. *Oncology Reports*.

[61] Sampson JR, Dolwani S, Jones S (2003). Autosomal recessive colorectal adenomatous polyposis due to inherited mutations of MYH. *The Lancet*.

[62] Davidson NO (2007). Genetic testing in colorectal cancer: who, when, how and why. *The Keio Journal of Medicine*.

[63] Fearnhead NS, Britton MP, Bodmer WF (2001). The ABC of APC. *Human Molecular Genetics*.

[64] Rustgi AK (2007). The genetics of hereditary colon cancer. *Genes and Development*.

[65] Wu G, Richards CS, Wu W (2001). Detection of sequence variations in the adenomatous polyposis coli (APC) gene using denaturing high-performance liquid chromatography. *Genetic Testing*.

[66] Batra S, Valdimarsdottir H, McGovern M, Itzkowitz S, Brown K (2002). Awareness of genetic testing for colorectal cancer predisposition among specialists in gastroenterology. *The American Journal of Gastroenterology*.

[67] Wijnen JT, Vasen HFA, Khan PM (1988). clinical findings with implications for genetic testing in families with clustering of colorectal cancer. *The New England Journal of Medicine*.

[68] Soravia C, Berk T, Cohen Z (2000). Genetic testing and surgical decision making in hereditary colorectal cancer. *International Journal of Colorectal Disease*.

[70] Meuller J, Kanter-Smoler G, Nygren AOH (2004). Identification of genomic deletions of the APC gene in familial adenomatous polyposis by two independent quantitative techniques. *Genetic Testing*.

[71] American Gastroenterological Association, Department of Health and Ageing

[72] Michils G, Tejpar S, Thoelen R (2005). Large deletions of the APC gene in 15% of mutation-negative patients with classical polyposis (FAP): a Belgian study. *Human Mutation*.

[73] Aaltonen LA, Salovaara R, Kristo P (1998). Incidence of hereditary non polyposis colorectal cancer and the feasibility of molecular screening for the disease. *The New England Journal of Medicine*.

[74] Hampel H, Frankel WL, Martin E (2005). Screening for the Lynch syndrome (hereditary non polyposis colorectal cancer). *The New England Journal of Medicine*.

[75] Vilar E, gruber SB (2010). Microsatellite instability in colorectal cancer-the stable evidence. *Nature Reviews Clinical Oncology*.

[76] Ho SMY, Ho JWC, Bonanno GA, Chu ATW, Chan EMS (2010). Hopefulness predicts resilience after hereditary colorectal cancer genetic testing: a prospective outcome trajectories study. *BMC Cancer*.

[77] Center MM, Jemal A, Ward E (2009). International trends in colorectal cancer incidence rates. *Cancer Epidemiology Biomarkers and Prevention*.

[78] Jemal A, Bray F, Center MM, Ferlay J, Ward E, Forman D (2011). Global cancer statistics. *CA: Cancer Journal for Clinicians*.

[79] Siegel RL, Jemal A, Ward EM (2009). Increase in incidence of colorectal cancer among young men and women in the United States. *Cancer Epidemiology Biomarkers and Prevention*.

[80] El-Sebai I (1961). Advanced bilharzial intestinal manifestations. The relation to cancer. *Kasr El-Aini Journal of Surgery*.

[81] Amano J (1981). Clinico pathological studies on the gastrointestinal schistosomiasis. *Japanese Journal of Parasitology*.

[82] Ming-Chai C, Chi-Yuan C, Pei-Yu C, Jen-Chun H (1980). Evolution of colorectal cancer in schistosomiasis. Transitional mucosal changes adjacent to large intestinal carcinoma in colectomy specimens. *Cancer*.

[83] Dimmette RM, Elwi AM, Sproat HF (1956). Relationship of schistosomiasisto polyposis and adenocarcinoma of large intestine. *The American Journal of Clinical Pathology*.

[84] Rutter M, Saunders B, Wilkinson K (2004). Severity of inflammation is a risk factor for colorectal neoplasia in ulcerative colitis. *Gastroenterology*.

[85] Brentnall TA, Haggitt RC, Rabinovitch PS (1996). Risk and natural history of colonic neoplasia in patients with primary sclerosing cholangitis and ulcerative colitis. *Gastroenterology*.

[86] Askling J, Dickman PW, Karlén P (2001). Family history as a risk factor for colorectal cancer in inflammatory bowel disease. *Gastroenterology*.

[87] Heuschen UA, Hinz U, Allemeyer EH (2001). Backwash ileitis is strongly associated with colorectal carcinoma in ulcerative colitis. *Gastroenterology*.

[88] Velayos FS, Loftus EV, Jess T (2006). Predictive and protective factors associated with colorectal cancer in ulcerative colitis: a case-control study. *Gastroenterology*.

[89] Itzkowitz SH, Harpaz N (2004). Diagnosis and management of dysplasia in patients with inflammatory bowel diseases. *Gastroenterology*.

[90] Medicare Australia

[92] Rex

[93] Hong SN, Choe WH, Lee JH (2012). back-to-back trial evaluating the usefulness of i-SCAN in screening colonoscopy. *Gastrointestinal Endoscopy*.

[94] Bianco MA, Cipolletta L, Rotondano G (2010). Prevalence of nonpolypoid colorectal neoplasia: an Italian multicenter observational study. *Endoscopy*.

[95] Kudo S, Tamura S, Nakajima T, Yamano H, Kusaka H, Watanabe H (1996). Diagnosis of colorectal tumorous lesions by magnifying endoscopy. *Gastrointestinal Endoscopy*.

[96] http://www.google.com/url?sa=t&rct=j&q=&esrc=s&frm=1&source=web&cd=1&ved=0CFMQFjAA&url=http%3A%2F%2Fwww.curetoday.com%2Findex.cfm%2Ffuseaction%2Farticle.PrintArticle%2Farticle_id%2F167&ei=mDPVT9eNAtDn8QP1ovSjAw&usg=AFQjCNHFYcyCUIU9jvvWfdhk4cwa03UPEA.

[97] Vasen HFA (2007). The Lynch syndrome (hereditary nonpolyposis colorectal cancer). *Alimentary Pharmacology and Therapeutics*.

[98] Grady WM (2005). Molecular basis for subdividing hereditary colon cancer?. *Gut*.

[99] National Hospital Cost Data Collection (2010). *Cost Report Round 13 (2008-2009)*.

[100] Nugent FW, Haggitt RC, Gilpin PA (1991). Cancer surveillance in ulcerative colitis. *Gastroenterology*.

[101] Bishop J, Glass P, Tracey E (2008). *Health Economics Review of Bowel Cancer Screening in Australia*.

[102] Australian Institute of Health and Welfare

[103] Cancer Institute NSW Monograph

[104] Platell C, Salama P, Barwood N, Makin G (2005). Performing a colonoscopy 12 months after surgery for colorectal neoplasia. *Australian and New Zealand Journal of Surgery*.

[105] Hassan C, Gaglia P, Zullo A (2006). Endoscopic follow-up after colorectal cancer resection: an Italian multicentre study. *Digestive and Liver Disease*.

[106] Hyman N, Moore J, Cataldo P, Osler T (2010). The high yield of 1-year colonoscopy after resection: Is it the handoff?. *Surgical Endoscopy and Other Interventional Techniques*.

[107] Aste H, Saccomanno S, Pugliese V, Santi L (1982). Colonoscopy in patients previously submitted to partial colectomy for colorectal cancer. *Acta Endoscopica*.

[108] Hong SN, Choe WH, Lee JH (2012). Prospective, randomized, back-to-back trial evaluating the usefulness of i-SCAN in screening colonoscopy. *Gastrointestinal Endoscopy*.

[109] American Cancer Society

[110] World Cancer Research Fund/American Institute for Cancer Research (2007). *Food, Nutrition, Physical Activity, and the Prevention of Cancer: A Global Perspective*.

[111] Braithwaite D, Emery J, Walter F, Prevost AT, Sutton S (2004). Psychological impact of genetic counseling for familial cancer: a systematic review and meta-analysis. *Journal of the National Cancer Institute*.

[112] Miles A

[113] Liljegren A, Lindgren G, Brandberg Y (2004). Individuals with an increased risk of colorectal cancer: perceived benefits and psychological aspects of surveillance by means of regular colonoscopies. *Journal of Clinical Oncology*.

[114] Gearhart SL, Ahuja N (2011). *Early Diagnosis and Treatmentof Colorectal Cancer*.

